# Effect of Non-Uniform Temperature Field on Sintering Performance of Conductive Silver Paste in Laser Sintering

**DOI:** 10.3390/ma18102358

**Published:** 2025-05-19

**Authors:** Wenkai Zu, Xingzhi Xiao, Tingting Liu, Mingfei Gu, Gang Li, Wenhe Liao

**Affiliations:** School of Mechanical Engineering, Nanjing University of Science and Technology, Nanjing 210094, China; zuwk@njust.edu.cn (W.Z.); liutingting@mail.njust.edu.cn (T.L.); mingfei@njust.edu.cn (M.G.); ligang@njust.edu.cn (G.L.); cnwho@mail.njust.edu.cn (W.L.)

**Keywords:** conductive silver paste, laser sintering, resistivity, non-uniform temperature field, effective sintering temperature

## Abstract

The non-uniform temperature field in laser sintering critically affects conductive silver paste performance, yet its quantitative relationship with sintering mechanisms remains unclear. This study addresses this issue by proposing effective sintering temperature (*T_a_*) and effective sintering time (*S_a_*) as metrics to link laser parameters and sintering temperature field with sintering performance. Through full-factorial experiments, finite element simulation, and in situ thermal monitoring, it was revealed that (1) Increasing laser power and reducing laser scanning speed effectively reduce resistivity. For example, at 10 W and 0.1 mm/s, the resistivity reached 6.81 μΩ·cm, which was 88.9% lower than the value of 61.11 μΩ·cm at 2 W and 0.5 mm/s. (2) The resistivity exhibits a threshold effect in its reduction across low-power (<3 W), medium-power (3~4 W), and high-power (>5 W) ranges. (3) The action of laser sintering parameters on sintering performance through *T_a_* and *S_a_*. The resistivity decreases are correlated with *T_a_*, exceeding the exothermic peaks (*T*_1_ = 196 °C and *T*_2_ = 232 °C). Unlike prior qualitative analyses, this work quantifies how non-uniform temperature fields govern sintering through *T_a_* and *S_a_*, offering a quantitative method to analyze the temperature field’s effect on sintering performance.

## 1. Introduction

With the rapid development of microelectronics packaging/interconnection [1], the additive manufacturing of electronic circuits [2,3], and printed electronics [4,5], has gained extensive applications in both industrial production and the scientific research domain. Meanwhile, due to its excellent electrical conductivity, flexibility, and printability [6], it exhibits broad application prospects in the field of flexible electronics, such as flexible sensors [7], wearable electronic devices [8], curved antennas [9], etc. How to achieve its rapid and high-quality sintering after printing has become the focus of current research [10,11]. Currently, sintering technologies for conductive silver paste encompass thermal sintering [12], laser sintering [13], xenon lamp sintering [12], microwave sintering [14], and chemical sintering [15]. Among these, laser sintering has garnered significant attention due to its point heat source, narrow heat-affected zone, and rapid sintering, which meet the requirements for in situ high-quality interconnect circuits manufacturing [16,17].

The distribution of the temperature field will have a significant impact on the sintering performance of conductive silver paste [11,18]. In laser sintering, the conductive silver paste absorbs light from the point laser source to generate heat, thereby sintering the conductive silver paste [19], and this process shows that laser sintering has the characteristics of transient, point heat source, and non-uniform heating [1], making its temperature field distribution different from uniform heating methods such as thermal sintering. Existing temperature field detection technologies, such as infrared thermography, infrared cameras, and thermocouples, are suitable for measuring the surface temperature of conductive silver paste during laser sintering [20], but accurately measuring the non-uniform temperature field distribution inside the conductive silver paste during the sintering process remains challenging.

With the development of numerical simulation techniques, finite element analysis (FEA) can be utilized to investigate the temperature field evolution during laser sintering processes. Zhao et al. [16] simulated the influence of laser sintering parameters on the maximum temperature attained in 20-μm-thick silver conductive ink during laser sintering and compared it to infrared thermography detection results to verify the feasibility of the simulation. Based on theoretical calculations, Paeng et al. [19] conducted theoretical calculations to investigate the penetration depth of different wavelengths in conductive silver paste, and their findings revealed that the direct laser penetration depth was less than 200 nm, while sintering at greater depths relies on the thermal energy converted from laser energy. According to finite element simulation, Skylar-Scott et al. [21] calculated the heat transfer distance along the circuit length direction during infrared laser sintering of conductive silver paste, demonstrating that the thermal energy propagated approximately 2 mm from the laser spot. Fu [22] found that, during laser sintering of 32-μm-thick silver conductive ink, the maximum temperature occurred at the top surface of the circuit, and the lowest temperature occurred at the bottom interface, with the temperature decreasing along the depth direction. Currently, the technology for analyzing the temperature field during laser sintering of conductive silver paste through finite element simulation is relatively mature, but existing research predominantly focused on the qualitative characterization of temperature field distribution patterns, with limited quantitative research on the effect of non-uniform temperature fields on the sintering performance of conductive silver paste.

This study aims to establish the correlation among laser process parameters, temperature field distribution, and sintering performance and quantitatively analyze the characteristics of non-uniform temperature fields in laser sintering to further reveal the influence mechanism of temperature fields on the sintering performance of conductive silver paste. First, a full-factor experiment is performed to clarify the influence patterns of laser process parameters on the resistivity and microstructure. Secondly, based on finite element simulation of the laser sintering temperature field and validated by comparing with in situ temperature monitoring results, a reliable laser sintering temperature field distribution is established. Thirdly, the distribution characteristics of the laser sintering temperature field are extracted and define two key sintering parameters: effective sintering temperature (*T_a_*) and effective sintering time (*S_a_*). Finally, the correlation among process parameters, sintering temperature field, and sintering performance is constructed to reveal the mechanism by which the laser sintering temperature field affects the sintering performance of conductive silver paste.

Unlike prior studies that focused on qualitative descriptions of temperature effects, this study provides a quantitative method to analyze the temperature field’s effect on sintering performance by using *T_a_* and *S_a_* as the metrics. It establishes a quantitative relationship among sintering parameters (e.g., laser power and scanning speed), sintering temperature fields (characterized by non-uniform distributions), and sintering performance (e.g., resistivity and porosity). This provides support for the manufacturing of low-resistivity conductive circuits, enabling better application of laser-sintered conductive circuits in flexible electronic product manufacturing and integrated structural circuit manufacturing. This theoretical framework can provide a universal method for optimizing the sintering process of other materials.

## 2. Influence of Process Parameters on Sintering Performance

### 2.1. Experimental Design

Full-factorial experiments were conducted on a custom-modified three-axis circuit printing platform equipped with direct writing deposition capability and an infrared continuous-wave laser (wavelength 808 nm, spot diameter 190 μm, and focal length 105 mm). The laser power is precisely tunable from 0 to 11 W with a resolution of 0.01 W. The laser scanning speed is software-controlled with a resolution of 0.01 mm/s. Adjusting the height of the collimator ensures that the silver conductive circuit is in the focal plane of the laser beam for sintering. The printer is equipped with a CCD camera (resolution: 3088 × 2064, pixel size: 2.4 μm × 2.4 μm, and frame rate: 60 fps), enabling visual programming of the direct writing nozzle and laser trajectory. The printer and software (LX-1.0.35-N) were TZ-DJ3315RCCD (Shenzhen Peach Automation Technology Co., Ltd., Shenzhen, China).

**Figure 1:**
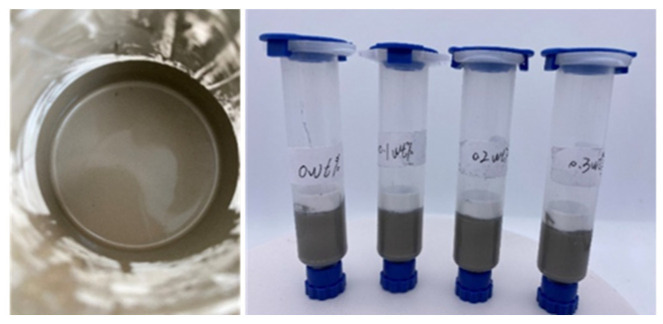
Self-formulated conductive silver paste.

The material was self-formulated high-viscosity conductive silver paste and formulated with 45 wt% micron-sized silver flakes, 45 wt% nano-silver particles, and 10 wt% deionized water, which were homogenized via planetary mixing. The diameter of the micron-sized silver flakes is 1~2 μm, and the diameter of the nano-silver particles (PAA-Ag) coated with polyacrylic acid on their surface is approximately 30 nm. The physical appearance of the silver paste material is shown in Figure 1.

**Figure 2:**
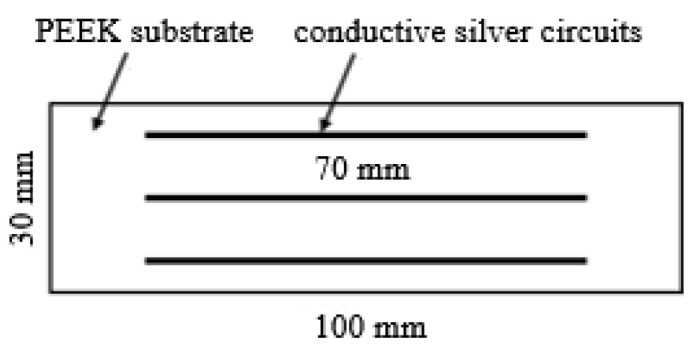
Designed experimental specimens.

Polyetheretherketone (PEEK) substrates fabricated by fused deposition modeling (FDM) were used, with dimensions of 100 mm (length) × 30 mm (width) × 5 mm (thickness). The FDM equipment was FUNMAT PRO 610HT (INTAMSYS, Shanghai, China). Conductive silver circuits were direct write printed onto the substrates, with three parallel lines deposited per substrate at a pitch of 10 mm. The manufacturing parameters of both the FDM-printed substrates and direct writing conductive silver circuits are shown in Table 1, with the corresponding experimental specimen depicted in Figure 2.

Through CCD visualization programming, the laser spot is focused on the deposited conductive silver paste circuit. The laser power range was determined as 2~10 W because (i) lower power (<2 W) could not provide sufficient sintering temperature, while (ii) higher power (>10 W) would melt the substrate. The laser scanning speed range was set to be 0.1~0.5 mm/s, since (i) higher speeds (>0.5 mm/s) resulted in inadequate energy input, degrading the sintering quality, whereas (ii) lower speeds (<0.1 mm/s) led to unacceptable process inefficiency.

**Figure 3:**
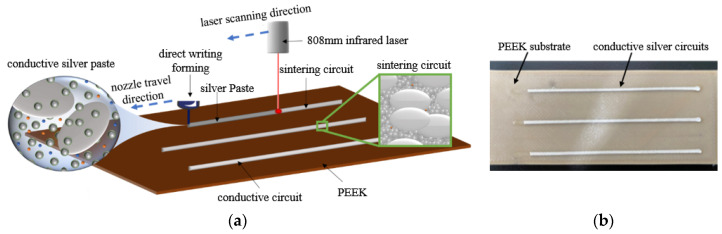
The process and sample of conductive circuits by laser sintering [23]: (**a**) experimental principle; (**b**) experimental sample after laser sintering.

With fixed laser defocusing distance (Δz) and sintering times, the laser power and laser scanning speed were selected as variables. The experiment employed 9 power levels and 5 speed levels, resulting in a total of 45 parameter combinations. Each conductive circuit was sintered only once, and three conductive circuits were prepared for each parameter combination. The resistivity of each parameter combination was calculated as the average of the three circuits to ensure reliability of the results. The specific experimental parameters are shown in Table 2, and the experimental principle and samples are illustrated in Figure 3.

### 2.2. Characterization Methods

The resistance of conductive circuits was measured using a DC low-resistance tester (HPS2512B, HELPASS, Kunshan, China), while their cross-sectional area was characterized by an industrial computerized imaging system (Micro-CT, FF35, YXLON, Hamburg, Germany). The resistivity of the conductive circuits was calculated using the following formula:

(1) $$\rho_{i}=R_{i}\times A_{i}/L$$

where *R_i_* and *A_i_* stand for the measured resistance and cross-section area of circuit sample *i*, respectively. The average resistivity of the three samples under each parameter was calculated.

The real-time laser sintering temperature was recorded using the infrared thermal (IR) camera (A615, FLIR, Portland, OR, USA). The accuracy of the IR camera was ±2 °C, and the object temperature range was set as +100 to +650 °C. The microstructure was characterized using Micro-CT, scanning electron microscopy (SEM, GeminiSEM 300, ZEISS, Oberkochen, Germany), and transmission electron microscopy (TEM, JEM 2100 F, JEOL, Tokyo, Japan).

### 2.3. Influence of the Laser Sintering Process on Resistivity

**Figure 4:**
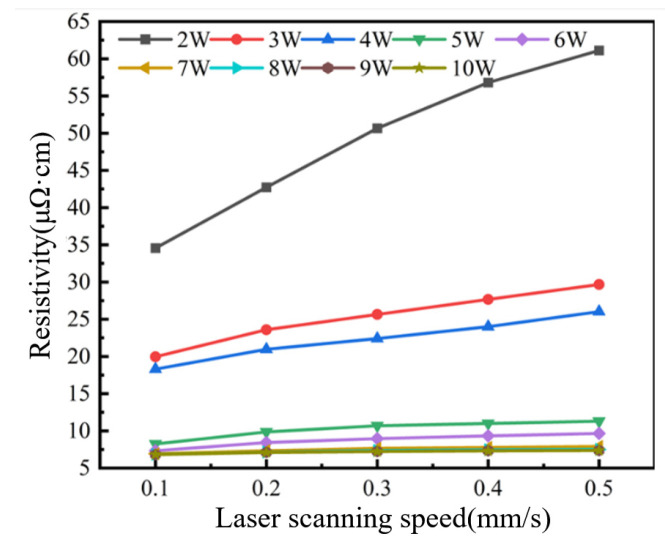
Resistivity evolution of conductive circuits versus laser scanning speed.

The resistivity of conductive circuits under different laser processing parameters was calculated, as shown in Figure 4. First, the effect of laser scanning speed on the resistivity of conductive circuits was analyzed. At each fixed laser power level, the resistivity showed an increasing trend with the increase in scanning speed. At a fixed 2 W laser power, the resistivity reached its maximum value of 61.11 μΩ·cm with a scanning speed of 0.5 mm/s but decreased to 34.56 μΩ·cm when the speed was reduced to 0.1 mm/s. Similarly, at 10 W power with 0.5 mm/s speed, the resistivity measured 7.35 μΩ·cm, which further reduced to 6.81 μΩ·cm when the speed was lowered to 0.1 mm/s under constant power (10 W).

**Figure 5:**
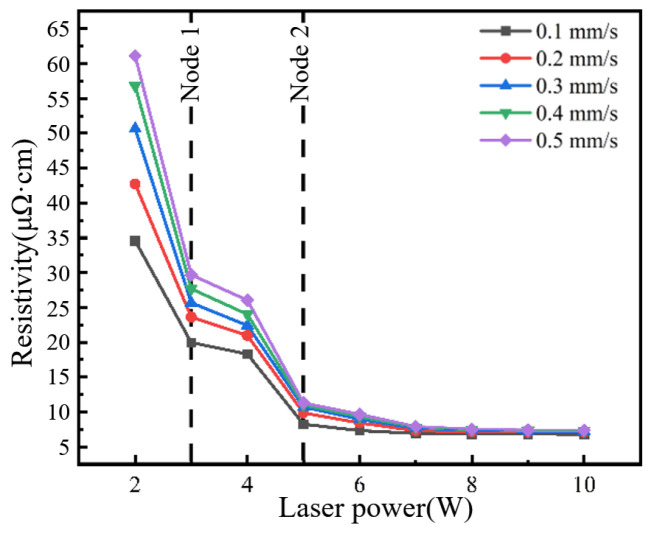
Resistivity evolution of conductive circuits versus laser power.

Figure 5 illustrates the effect of laser power on resistivity. Across all tested scanning speeds, the resistivity of the circuits exhibited a consistent decreasing trend with increasing laser power. As evidenced by the prior analysis of scanning speed effects, the 0.1 mm/s condition yielded optimal resistivity values. Therefore, we specifically analyzed the influence of laser power on resistivity at the scanning speed of 0.1 mm/s. Based on the slope variation of the resistivity versus laser power in Figure 5, the influence of laser power on resistivity was divided into three regimes by two critical transition points at 3 W and 5 W:

- Low-power regime (<3 W): resistivity was 34.56 μΩ·cm;
- First transition (3 W): resistivity abruptly decreased by 42.3% to 19.96 μΩ·cm;
- Medium-power regime (3~4 W): resistivity exhibited marginal variation within 18.30~19.96 μΩ·cm (−8.3% variation);
- Second transition (5 W): resistivity dropped sharply by 55.0% to 8.23 μΩ·cm;
- High-power regime (>5 W): absolute value of the resistivity stabilized with minimal fluctuation within 6.81~8.23 μΩ·cm (−17.3% variation).

**Figure 6:**
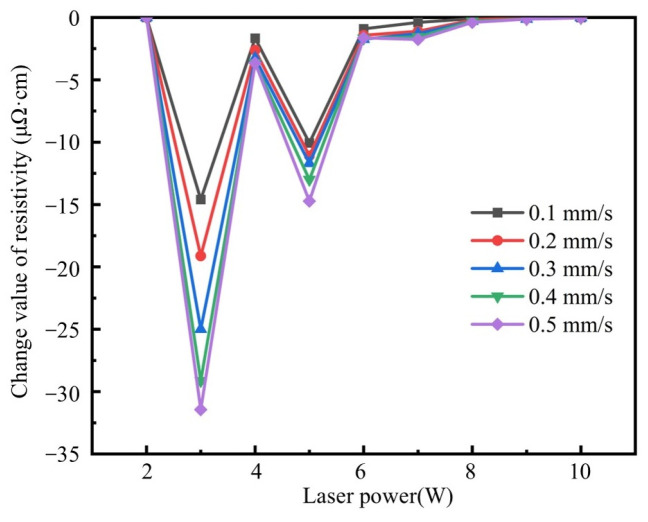
The change in value of resistivity per 1 W increase in laser power.

Figure 6 quantitatively demonstrates the effect of laser power variation on resistivity. The resistivity reduction per 1 W power increment reflects the efficacy of power elevation, where diminishing absolute values indicate progressively weaker influence of laser power enhancement on resistivity reduction. The results demonstrated significant resistivity reduction when increasing the laser power from 2 W to 3 W (−14.60 μΩ·cm) and from 4 W to 5 W (−10.07 μΩ·cm). However, the power-enhancement effect diminished substantially at the laser power above 6 W, particularly beyond 8 W. For instance, at a scanning speed of 0.1 mm/s, the laser power increased from 9 W to 10 W, and the resistivity only reduced from 6.82 μΩ·cm to 6.81 μΩ·cm (−0.01 μΩ·cm). This trend suggests that, under low-speed (0.1 mm/s) and high-power (10 W) conditions, the conductive silver paste achieves near-complete sintering, yielding the minimum resistivity of 6.81 μΩ·cm. Further reduction in resistivity through additional decreases in scanning speed and an increase in laser power is inefficient.

### 2.4. Influence of Laser Power on the Microstructure

**Figure 7:**
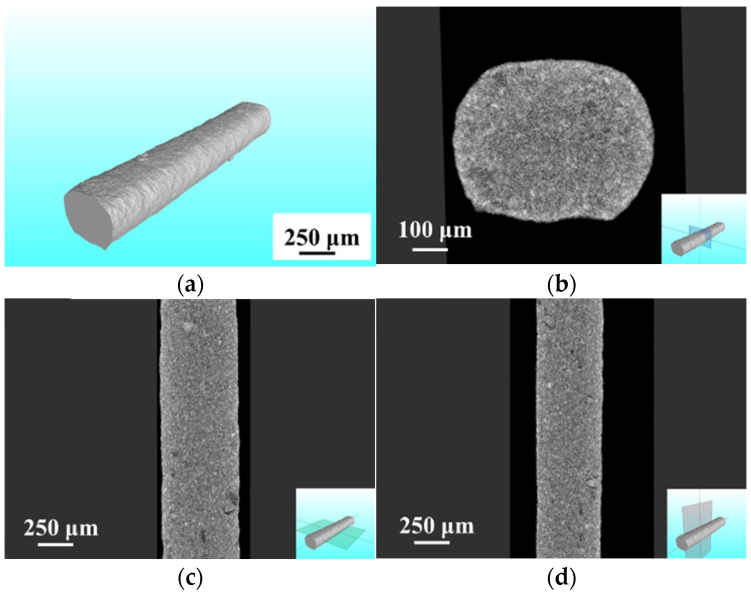
Overall morphology of the conductive circuit microstructure (CT): (**a**) 3D morphology; (**b**) cross-section morphology; (**c**) width direction morphology; (**d**) height direction morphology.

This section investigates the influence of laser power on the microstructure and organic residue content of sintered conductive circuits at the optimal scanning speed of 0.1 mm/s. Thus, a deeper understanding of the influence pattern of laser power on resistivity can be gained. Figure 7 displays the overall morphology of the sintered conductive circuit. Figure 7a presents a three-dimensional (3D) CT-reconstructed image of the conductive circuit fabricated at a laser power of 10 W, showing no significant macroscopic pores on its external surface. Figure 7b–d exhibit cross-sectional views of Figure 7a along three distinct orientations, revealing the internal morphology. The results demonstrated the absence of observable macroscopic voids in all three cross-sectional directions. However, variations in grayscale contrast across the cross-sectional images suggest the persistence of microscopic porosity within the conductive circuit, indicating incomplete structural homogeneity and densification.

**Figure 8:**
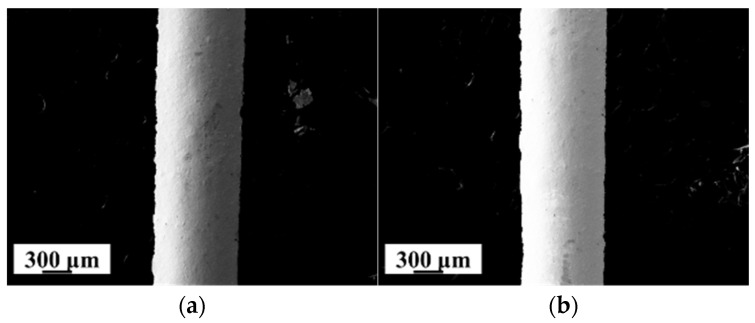

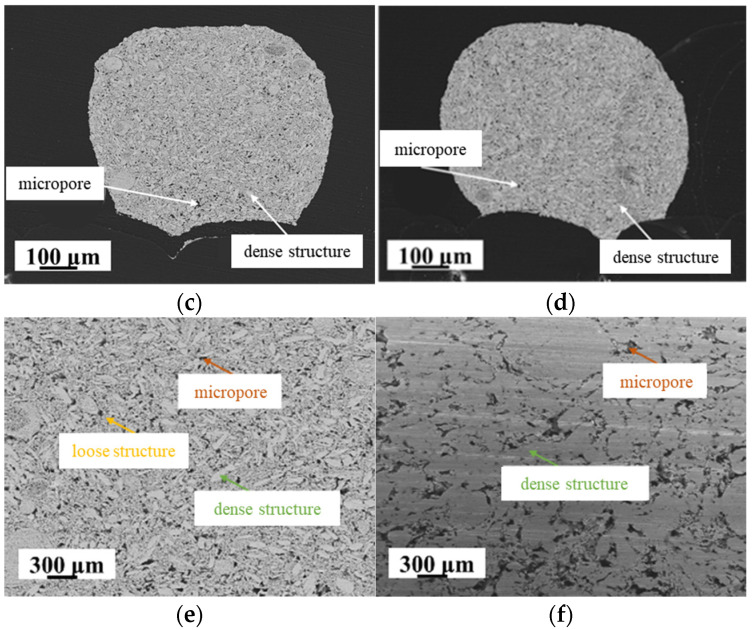
Overall morphology of the conductive circuit microstructure (SEM): (**a**) upper surface (2 W); (**b**) upper surface (10 W); (**c**) cross-section (2 W); (**d**) cross-section (10 W); (**e**) micromorphology (2 W); (**f**) micromorphology (10 W).

Figure 8 presents the SEM image of the conductive circuits fabricated at laser powers of 2 W and 10 W. Figure 8a,b display the surface morphology of the circuits, revealing no observable defects on their external surfaces. The cross-sectional morphologies in Figure 8c,d indicate that there are no significant differences in the overall microstructure of the circuits. This observation aligns with the CT analysis results in Figure 7, confirming the absence of macroscopic pores within the conductive circuits [24]. Irregularly distributed locally dense structures are interspersed across the cross-section of the conductive circuit, with these localized dense structures having a higher density than the surrounding areas. Additionally, micropores are randomly dispersed on the cross-section of the conductive circuit. The size, proportion of these localized dense structures, and micropores within the conductive circuit may be the cause of the differences in resistivity under different sintering parameters. As shown in Figure 8e,f, the microstructures of the circuit can be classified into three categories: (1) localized dense structures, which have a higher density than the surrounding regions; (2) micropores; and (3) loose structures, composed of incompletely fused silver particles. When the laser power was 2 W, a large number of small-sized, local, dense structures were distributed within the conductive circuit, with pores and loose structures interspersed among them. As the laser power increased to 10 W, the size of these dense structures grew, and the loose structures composed of incompletely fused particles completely disappeared, leaving the conductive circuit internally composed of localized dense structures and micropores.

**Figure 9:**
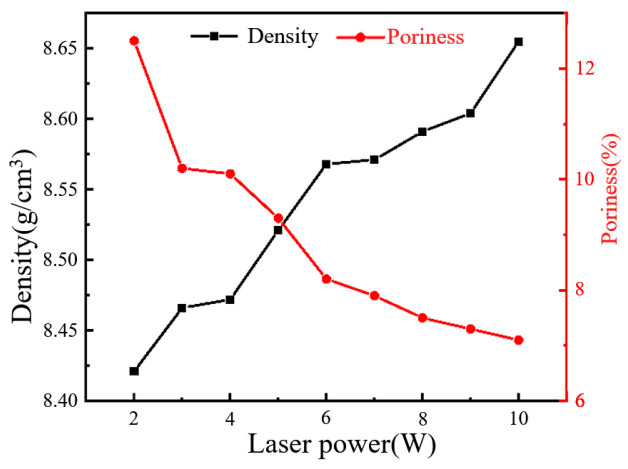
Density and porosity under different laser powers.

Figure 9 presents the density and porosity of laser-sintered conductive circuits measured by Archimedes’ method, providing further evidence for understanding the laser power-dependent evolution of the internal porosity within conductive circuits. With the increase in laser power, the density of the circuits keeps rising, while the porosity steadily decreases. When the laser power is 2 W, the density is 8.421 g/cm^3^; when the laser power is 10 W, the density increases to 8.655 g/cm^3^ (closely matching the density of bulk silver at 10.5 g/cm^3^). The measurement results of porosity indicate that, when the laser power was 2 W, the sintered circuit porosity was 12.5%; when the laser power was 10 W, the sintered circuit porosity reduced to 7.1%. This trend demonstrates that, as the laser power increases, the silver particles in the conductive silver paste melt more thoroughly, leading to a progressive reduction in the internal porosity.

## 3. Influence of the Process Parameters on Temperature Field Distribution

### 3.1. Analysis of the Laser Action Mechanism

The interaction process of the laser with conductive silver paste is a typical photothermal process. That is, when the laser interacts with the substances within the silver paste, the light energy is absorbed by the substances and converted into thermal energy. This process causes an increase in the temperature of the substances, leading to changes in their physical or chemical properties, thus achieving sintering. Therefore, the mechanism of laser action can be categorized into two regimes. One is the direct sintering, in which the laser directly penetrates at a certain depth, and the other is the thermal sintering caused by heat transfer.

Laser penetration depth (*d_p_*) is the depth at which the intensity of a laser incident on the surface of a material attenuates to 1/*e* (about 37%) of its original intensity, where *e* is the base of the natural logarithm [25]. *d_p_* can be calculated by the following formula:

*d_p_* = 1/α (2)

where α is the material’s absorption coefficient with units of m^−1^. Based on the literature data [19], the absorption coefficient of the conductive silver paste for the 808 nm laser wavelength used in this study was estimated as 6 × 10^6^ m^−1^, yielding a calculated *d_p_* of approximately 167 nm.

**Figure 10:**
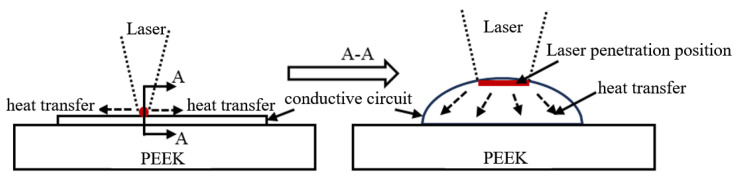
Schematic diagram of the action mechanism of laser heat on conductive silver paste.

The thickness of the conductive circuit formed in this paper is above 100 μm, which far exceeds the *d_p_*. When the laser acts on the surface of the conductive silver paste, only the top layer (about 200 nm deep) is directly affected by the laser, while the remaining part of the circuit relies on the thermal energy generated by the laser’s photothermal effect for sintering. Meanwhile, along the direction of the conductive circuit, the thermal energy converted from the laser also sinters the silver paste through heat transfer. The mechanism of the laser action is shown in Figure 10. Based on the understanding of this process, it can be seen that the laser sintering of the silver paste is essentially a thermal sintering process, and the influence of the laser parameters on the sintering performance is exerted via the temperature field. Therefore, it is crucial to study the temperature field distribution during the laser sintering process.

### 3.2. Temperature Field Monitoring and Simulation in Laser Sintering

**Figure 11:**
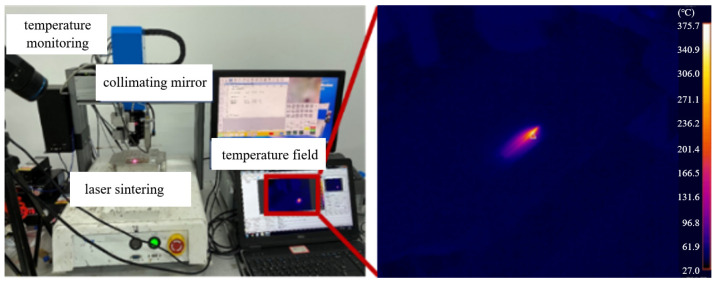
The monitoring of the sintering temperature of conductive circuits.

**Figure 12:**
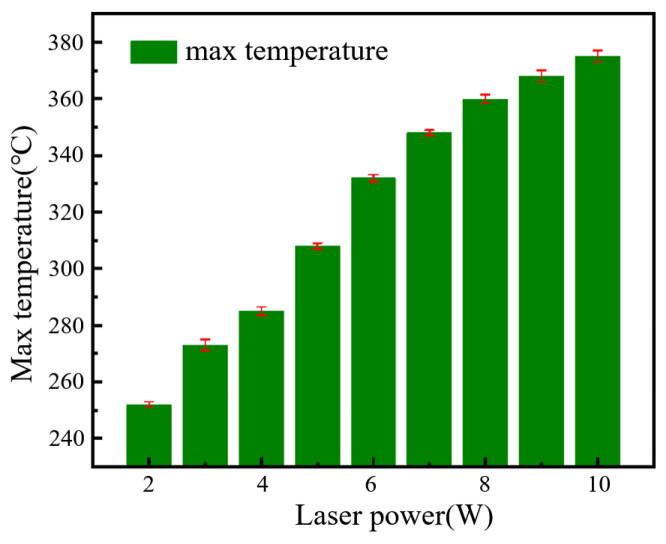
Maximum surface temperatures at varying laser powers.

A thermal infrared imager was utilized to monitor the temperature changes on the surface of conductive circuits during sintering, as shown in Figure 11. The accuracy of the thermal infrared imager was ±2 °C, and the object temperature range was set as +100 to +650 °C. The thermal infrared imager measurement corrections were taken by comparing to the standard objects’ temperature test results, which were measured by a contact thermocouple. Figure 12 lists the maximum surface temperatures when the laser power is different at 0.1 mm/s for the laser scanning speed when sintering conductive silver paste. As the laser power increased, the maximum surface temperature on the conductive circuits continuously rose. When the laser power is 2 W, the monitored maximum surface temperature is 252 °C; with the laser power set to 10 W, the monitored maximum surface temperature increases to 375 °C.

**Figure 13:**
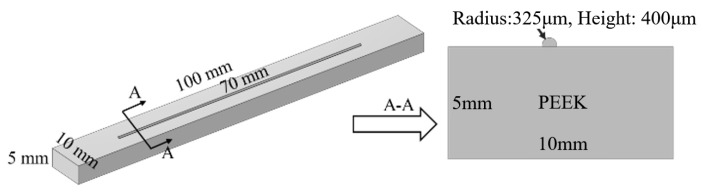
Finite element simulation model of laser sintering for the conductive circuit.

Due to the limitations of the experimental methods, temperature monitoring can only be achieved on the surface of the circuit, making it impossible to obtain the temperature field distribution throughout the entire conductive circuit. Therefore, finite element simulation was used to model the laser sintering process of silver paste to analyze the temperature field changes inside the conductive circuit during the entire sintering process. Based on transient thermal analysis, a three-dimensional model consistent with the actual sample size was constructed, as shown in Figure 13. The grid for the conductive circuit used a quadrilateral grid with a cell size of 100 μm (slightly larger than the laser radius, 95 μm). While the grid for the substrate used conventional quadrilateral grids, the total number of cells was 32,601, and the average unit mass of skew was 0.8424.

**Figure 14:**
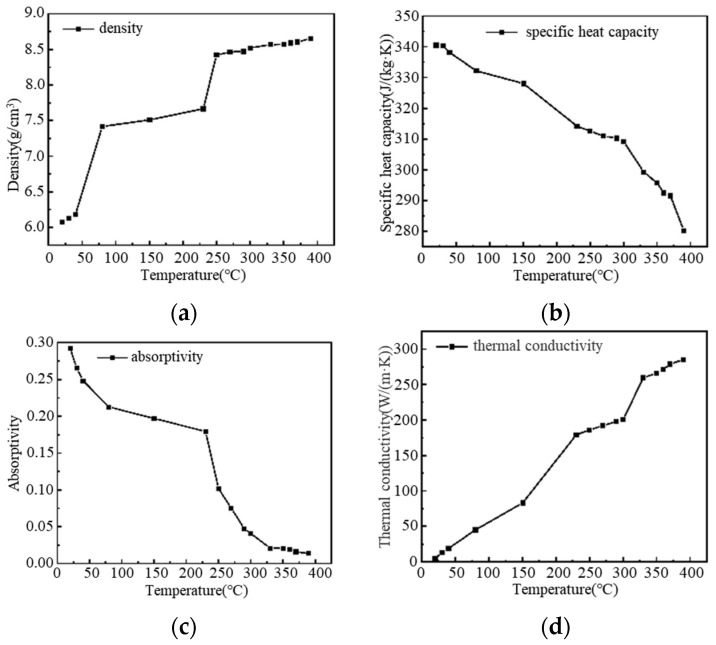
Material properties of silver paste obtained by experiment: (**a**) density at different temperatures; (**b**) specific heat capacity at different temperatures; (**c**) absorptivity at different temperatures; (**d**) thermal conductivity at different temperatures.

The substrate material was defined as PEEK from the software’s built-in material library, while the conductive circuit material was defined as a user-customized silver paste. For the silver paste material, during the laser sintering process, the solvent evaporation, organic removal, and silver particle fusion are irreversible processes. Therefore, its related material properties (e.g., density, laser absorption rate, specific heat capacity, and thermal conductivity) are also irreversible. They only evolve with the increase in temperature and remain unchanged upon cooling. The following state selection variables are defined in the program to control this process:

(3) $$M=\left\{\begin{array}{l} M_{t-1}\;\;\;\;\;\;if\;(T_{t}<T_{t-1})\\ M_{t}\;\;\;\;\;\;\;\;\;if\;(T_{t}\geq T_{t-1}) \end{array}\right.$$

where *M* represents the properties of the silver paste material, such as density and specific heat capacity; *M_t_*_−1_ represents the material properties at the previous moment; *M_t_* represents the material properties at the current moment; *T* is the temperature; and *t* is the simulation moment. Based on experimental measurement results, including density, specific heat capacity, laser absorption rate, and thermal conductivity, the material properties of the silver paste are defined using interpolation functions, as shown in Figure 14.

The laser was set as a Gaussian surface light source, with the same parameters as the experiment, and the radius was 95 μm. It scanned along the upper surface centerline of the conductive circuit from the leftmost to the rightmost end at 0.1 mm/s. The simulation was conducted using the Solid Heat Transfer module, with the ambient temperature set to 20 °C. The PEEK substrate had thermal insulation on all surfaces except the upper surface. Considering the thermal convection between the upper surface of the PEEK substrate and the ambient environment, as well as the thermal radiation from the upper surface of the PEEK substrate and the surfaces of the circuit, the probe settings include:

- The maximum temperature within the conductive circuit domain (denoted as *T_max_*);
- The center point of the upper surface in the conductive circuit cross-section (denoted as *Z*_0_);
- A point 10 μm below the surface (denoted as *Z*_10_);
- A point 200 μm below the surface (denoted as *Z*_200_);
- A point 400 μm below the surface (i.e., the bottom surface, denoted as *Z*_400_);
- The edge point of the conductive circuit’s bottom surface farthest from the center of the upper surface (denoted as *Z_min_*).

**Figure 15:**
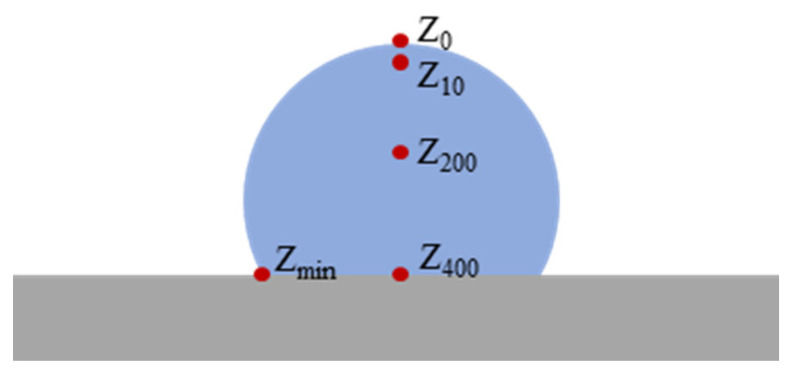
Simulation setting of the laser sintering temperature field.

These configurations are illustrated in Figure 15.

**Figure 16:**
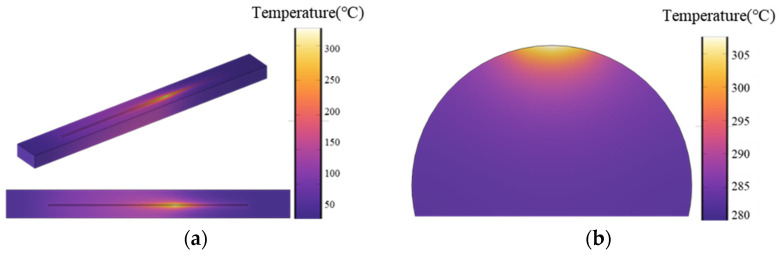
Simulation temperature field morphology of laser sintering: (**a**) upper surface temperature nephogram; (**b**) temperature nephogram of the conductive circuit cross-section.

The temperature field morphology obtained through simulation is shown in Figure 16. The upper surface temperature field morphology in Figure 16a is similar to that obtained from monitoring in Figure 11. Figure 16b shows the distribution of the temperature field across the cross-section of the circuit. The results indicate that the maximum temperature is at the center of the upper surface of the circuit, with the temperature field spreading out in a fan shape around this central point throughout the entire cross-section, which aligns with the analysis results of the cross-section in Figure 10.

**Figure 17:**
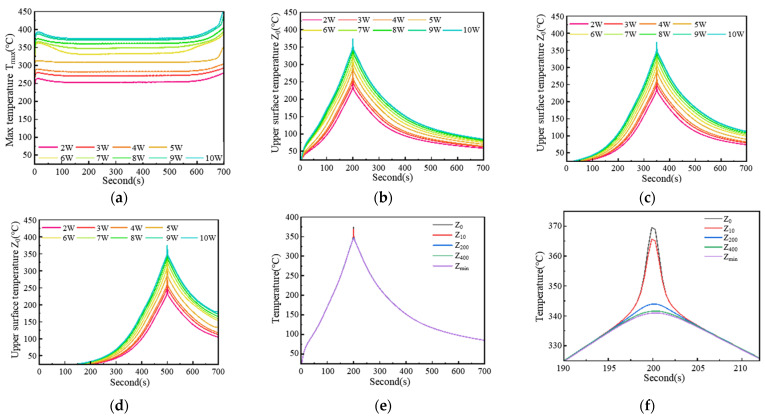
Simulation temperature of laser sintering for the conductive circuit: (**a**) maximum temperature of the conductive circuit; (**b**) upper surface at 20 mm (*Z*_0_); (**c**) upper surface at 35 mm (*Z*_0_); (**d**) upper surface at 50 mm (*Z*_0_); (**e**) different positions at 20 mm; (**f**) enlarged view of different positions at 20 mm.

The maximum internal temperature of the conductive circuit under different laser powers is shown in Figure 17a. As the laser is applied to the conductive silver paste and begins sintering, the maximum internal temperature of the conductive circuit quickly reaches a steady-state value, which corresponds to the experimental values obtained from monitoring in Figure 11. As summarized in Table 3, the simulated maximum temperatures exhibited excellent agreement with the experimental data, with deviations ranging from −4 °C to +2 °C (mean accuracy: 99.47%). Figure 17b–d display the temperature profiles at 20 mm, 35 mm, and 50 mm from the sintering start point, respectively. The results demonstrate consistency in both the maximum temperatures and the overall trend of temperature change on the upper surface at 20 mm, 35 mm, and 50 mm locations. In Figure 17a, the maximum temperature at any surface corresponds to the instant when the laser spot center aligns with that position. In order to achieve this maximum temperature, there is a heating process of approximately 20 s before the laser arrives, followed by a cooling process of approximately 50 s after the arrival of the laser.

Figure 17e shows the temperature changes at different positions along the cross-section, indicating that, during laser sintering, the same cross-section of the conductive circuit experiences consistent heating and cooling processes at different locations, with only temperature differences observed when the laser reaches each position. The magnified view in Figure 17f illustrates that, within approximately 10 s before and after the laser spot arrives (i.e., at the time 200 s), the temperature varies across different positions on the cross-section, with the peak temperature occurring at the edge of the bottom surface of the conductive circuit (*Z_min_*). This is because the maximum temperature forms at the center of the laser, while other parts rely on heat transfer to acquire thermal energy, resulting in the minimum temperature being achieved at the edge of the conductive circuit’s cross-section farthest from the center of the upper surface.

## 4. Quantitative Analysis of Temperature Field Effects on Sintering Performance

### 4.1. Relative Relationship Between Effective Sintering Temperature and Thermal Characteristics of Conductive Silver Paste

From the analysis of the temperature field in the previous section, it is known that the primary influence by which lasers affect conductive silver paste is through the absorption of light energy and its conversion into heat for sintering. However, unlike the thermal sintering process, the temperature field changes during sintering exhibit two characteristics:

- non-uniform heating. Unlike the uniform heating in thermal sintering, the top-down heat transfer process in laser sintering results in non-uniform heating across the cross-section of the conductive circuit during sintering.
- non-steady-state temperature changes. Due to the heat transfer along the direction of the conductive circuit, the sintering temperature at a point on the conductive circuit first rises and then falls, creating a preheating and cooling process.

Compared to the surface temperature measured directly by thermal imaging, the bottom edge exhibited the lowest cross-sectional temperature. Since all other locations within the circuit cross-section showed higher temperatures, it can be concluded that the sintering efficacy was weakest at the substrate-proximal interface. Therefore, this paper refers to the peak temperature at the bottom edge as the “effective sintering temperature” (denoted as *T_a_*). The *T_a_* values obtained from sintering simulations for various laser powers are shown in Table 4.

**Figure 18:**
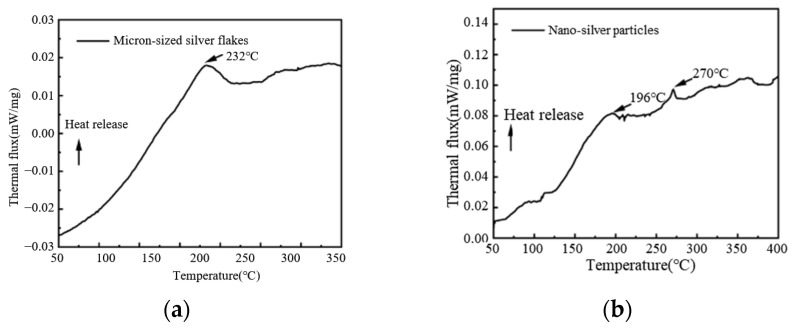
DSC curves of micron-sized silver flakes and nano-silver particles: (**a**) micron-sized silver flakes; (**b**) nano-silver particles.

The DSC curve in Figure 18 analyzes the thermal properties of the raw material powder. The peaks in the DSC curve represent a combination of exothermic and endothermic responses, originating from processes such as silver particle recrystallization and organic substance removal [26]. The exothermic reactions correspond to particle fusion, reduction of crystal defects, and particle recrystallization, while the endothermic reactions correspond to the thermal decomposition and desorption of organic matter. PAA-Ag shows exothermic peaks at 196 °C (denoted as *T*_1_) and 270 °C (denoted as *T*_3_), whereas micron-sized silver flakes exhibit an exothermic peak around 232 °C (denoted as *T*_2_).

**Figure 19:**
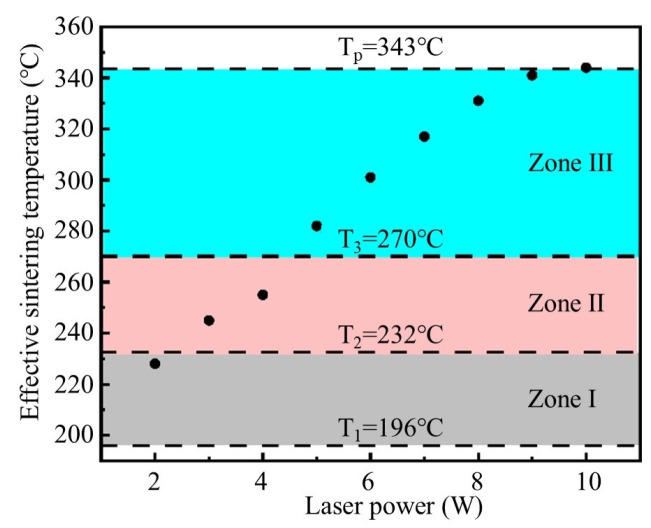
Effective sintering temperature zone based on exothermic peak temperatures.

The *T_a_* is categorized into three zones based on exothermic peak temperatures under varying laser powers, with the lower bound defined by the first exothermic peak temperature (*T*_1_ = 196 °C) of PAA-Ag and the upper bound set at the melting temperature (*T_p_* = 343 °C) of the PEEK substrate, as shown in Figure 19. The *T_a_* at different laser powers is divided into three zones.

- Zone I (*T*_1_ < *T_a_* < *T*_2_): When *T_a_* is between *T*_1_ and *T*_2_, the nano-silver particles initiate sintering, while the micron-sized silver flakes remain below the sintering temperature, resulting in partial consolidation of the conductive silver paste with suboptimal performance.
- Zone II (*T*_2_ < *T_a_* < *T*_3_): When *T_a_* is between *T*_2_ and *T*_3_, the sintering of the nano-silver particles intensifies, and the micron-sized silver flakes begin to sinter, thereby causing the sintering performance to improve compared to Zone I.
- Zone III (*T*_3_ < *T_a_* < *T_p_*): When *T_a_* is between *T*_3_ and *T_p_*, all conductive phases within the conductive silver paste can start to undergo full sintering, and the sintering effect is continuously enhanced with the increase in laser power.

**Figure 20:**
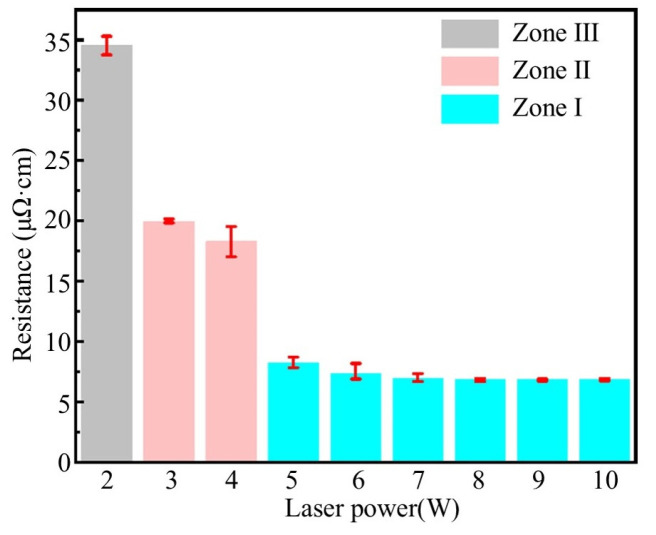
Relationship between the resistivity and effective sintering temperature interval.

Based on the effective sintering temperature range shown in Figure 19, we further analyzed the resistivity evolution under different laser powers to obtain the influence of laser power on the variation in resistivity through the effective sintering temperature, as illustrated in Figure 20. The results indicate that, when *T*_1_ < *T_a_* < *T*_2_, the conductive silver paste undergoes initial sintering, forming percolative conductive pathways while maintaining relatively high resistivity. When *T*_2_ < *T_a_* < *T*_3_, micron-sized silver flakes reach the sintering condition, leading to a rapid decrease in resistivity. As *T_a_* exceeds *T*_3_, both nanoscale silver particles and micron-sized silver flakes will thoroughly sinter, further reducing the resistivity to a lower level.

### 4.2. Influence of Effective Sintering Time on Sintering Performance

**Figure 21:**
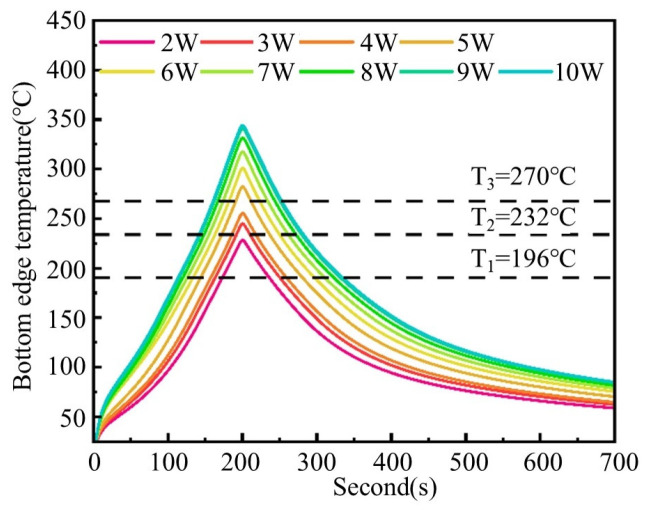
Definition of effective sintering time with different laser powers.

Unlike the steady-state temperature profile in conventional thermal sintering, laser sintering exhibits non-steady-state temperature changes. We quantitatively characterize the effective sintering process by defining the duration during which the sintering temperature at the edge of the bottom surface exceeds the exothermic peak temperatures of both nano-silver particles and micro-sized silver flakes as the effective sintering time (*S_a_*), as illustrated in Figure 21. The horizontal coordinate difference between the two intersection points of each temperature curve and the sintering peak temperature line represents the effective sintering time. The results indicate that, with the increase in laser power, the *S_a_* becomes longer, and the effective sintering intervals vary under different laser powers.

**Figure 22:**
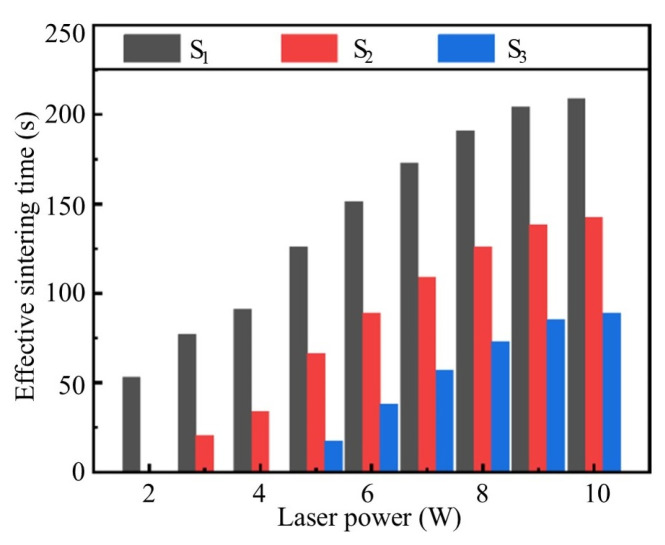
Effective sintering time (*S_a_*) at different laser powers.

The results of *S_a_* under different laser powers are shown in Table 5, with the variation trend illustrated in Figure 22. Based on the analysis of *S_a_*, the effective sintering time can be divided into three categories:

- *S*_1_: The duration when the sintering temperature exceeds *T*_1_ represents the period during which the sintering temperature remains above the first exothermic peak temperature of the nano-silver particles. Within this temperature range, the nano-silver particles undergo initial sintering.
- *S*_2_: The duration above temperature *T*_2_ represents the time period during which the sintering temperature exceeds the exothermic peak temperature of micron-sized silver flakes, enabling their effective sintering process.
- *S*_3_: The duration when the sintering temperature is greater than *T*_3_ represents the period when the sintering temperature exceeds the second sintering peak temperature of the nano-silver particles, ensuring thorough sintering of the nanoparticles.

The results show that, as the laser power increases, *S*_1_, *S*_2_, and *S*_3_ are all prolonged, enabling both nano-silver particles and micron-sized silver flakes to obtain sufficient sintering time.

According to the analysis of effective sintering temperature effects on resistivity in Figure 20, we can obtain the *T_a_* and *S_a_* of three laser power intervals, as shown in Table 6.

From Table 6, we can observe that, when the laser power increases from 2 W to 3 W, the *S*_2_ abruptly changes from 0 s to 21 s, and when the laser power increases from 4 W to 5 W, the *S*_3_ abruptly changes from 0 s to 17 s. It can be observed that, when *S_a_* transits from zero to a finite value, it can achieve a rapid decrease in inter-regional resistivity; as *S_a_* continues to prolong the rise of *T_a_*, it can lead to a gradual reduction in resistivity within the interval.

## 5. Conclusions

A full-factorial experiment was conducted to systematically investigate the effects of laser power and scanning speed on the resistivity and microstructure of sintered conductive circuits. Through integrated theoretical analysis, in situ monitoring, and sintering simulation, the temperature field distribution characteristics during laser sintering of conductive silver paste were elucidated. Through defining an effective sintering temperature (*T_a_*) and effective sintering time (*S_a_*), a quantitative analysis was conducted on the influence of laser-induced temperature fields on sintering performance. This established the correlation among the laser process parameters, temperature field distribution, and sintering performance.

1. Increasing laser power and reducing laser scanning speed can cause a decrease in the resistivity of conductive circuits. When the laser scanning speed is 0.1 mm/s and the laser power is 10 W, the minimum resistivity is 6.81 μΩ·cm. The laser scanning speed remains constant, the laser power is reduced to 2 W, and the resistivity will increase to 34.56 μΩ·cm. The laser power remains unchanged, the laser scanning speed increases to 0.5 mm/s, and the resistivity is 7.3 μΩ·cm.
2. The resistivity variation exhibited a stepwise decreasing trend across three laser power regimes: low-power (<3 W), medium-power (3~4 W), and high-power (>5 W), characterized by rapid inter-regime reduction and gradual intra-regime decline within each power regime. In the low-power range, the resistivity is 34.56 μΩ·cm, and the porosity is 12.5%. In the medium-power range, the resistivity ranges from 18.30 to 19.96 μΩ·cm, and the porosity ranges from 10.1% to 10.2%. In the high-power range, the resistivity is 6.81~8.23 μΩ·cm, and the porosity is 7.1~9.3%.
3. The laser sintering parameters determine the final sintering performance by influencing the temperature field through the effective sintering temperature and effective sintering time. Higher laser power elevates the effective sintering temperature beyond the exothermic peak of specific components, leading to rapid inter-regime resistivity reduction. Further increasing the laser power elevated the effective sintering temperature and extended the effective sintering time, thereby reducing the resistivity within the parameter regime and achieving superior sintering performance.

This provides a universal method for optimizing the sintering process of other materials. Through optimized parameter combinations, we achieved a minimum resistivity of 6.81 μΩ·cm. However, this value remains significantly higher than the theoretical resistivity of pure silver (1.65 μΩ·cm). To bridge this gap, innovative approaches, such as hybrid sintering techniques and machine learning, may be necessary to further reduce resistivity. Concurrently, while the lowest porosity attained was 7.1%, achieving higher-density conductive circuits demands advanced technologies to reduce the formation of pores. These dual challenges underscore the need for paradigm-shifting methodologies in laser-processed conductive circuitry. We hope to reduce the resistivity to near that of pure silver, so that the printed conductive lines can be better applied to flexible electronic product manufacturing and integrated structural circuit manufacturing. These are all challenges in printing conductive circuits with better performance.

## Figures and Tables

**Table 1:** Manufacturing parameters of FDM and direct writing forming.

Categories	Process	Parameters	Size
substrates (PEEK)	FDM	100% filled; print speed: 80 mm/s layer thickness: 0.2 mm	100 mm (length) × 30 mm (width) × 5 mm (thickness)
circuits	direct writing	nozzle diameter: 0.41 mm; print speed: 8 mm/s; extrusion pressure: 1.5 bar; nozzle height: 400 μm; distance of lines: 10 mm	70 mm (length) × 650 μm (width) × 400 μm (thickness)

**Table 2:** Experimental parameters setting of laser sintering.

Parameter Category	Parameter	Setting
Fixed parameters	Laser setting	focal length: 100 mm; Defocus distance: 0 mm
	Scanning passes (*n*)	*n* = 1
	Number of samples	3 samples for each parameter combination
Variable parameters	Laser power	2 W, 3 W, 4 W, 5 W, 6 W, 7 W, 8 W, 9 W, 10 W
	Scanning speed	0.1 mm/s, 0.2 mm/s, 0.3 mm/s, 0.4 mm/s, 0.5 mm/s

**Table 3:** Experimental value and simulation value of the maximum sintering temperature.

Laser power (W)	2	3	4	5	6	7	8	9	10
Experimental monitoring temperature (°C)	252	273	285	308	332	348	360	368	375
Sintering simulation temperature (°C)	253	271	283	309	328	347	361	370	376

**Table 4:** Effective sintering temperature (*T_a_*) at different laser powers.

Laser power (W)	2	3	4	5	6	7	8	9	10
Effective sintering temperature (°C)	228	245	255	282	301	317	331	341	346

**Table 5:** Effective sintering time (*S_a_*) at different laser powers.

Laser power (W)	2	3	4	5	6	7	8	9	10
*S*_1_ (s)	53	77	91	126	151	173	191	204	209
*S*_2_ (s)	0	21	34	66	89	109	126	138	143
*S*_3_ (s)	0	0	0	17	38	57	73	85	89

**Table 6:** Effective sintering time (*S_a_*) in different laser power regimes.

Laser Power Regimes	*T_a_* (°C)	*S*_1_ (s)	*S*_2_ (s)	*S*_3_ (s)
Low	228	53	0	0
Medium	245~255	77~91	21~34	0
High	282~346	126~209	66~143	17~89

## Data Availability

The original contributions presented in this study are included in the article. Further inquiries can be directed to the corresponding author

## Abbreviations

The following abbreviations are used in this manuscript:

FDM	Fused Deposition Modeling
PEEK	Polyetheretherketone
CCD	Charge-Coupled Device
CT	Computed Tomography
